# A Comparative Analysis of the Bleeding Profile and Quality of Life Among Women With Hemophilia Genotype Compared to Other Bleeding Disorders

**DOI:** 10.7759/cureus.68636

**Published:** 2024-09-04

**Authors:** Nita Radhakrishnan, Archit Pandharipande, Anukriti Srivastava, Shruti Verma, Eby P Baby, Hari M Gaire, Savitri Singh

**Affiliations:** 1 Department of Pediatric Hematology Oncology, Post Graduate Institute of Child Health, Noida, IND; 2 Department of Pathology, Post Graduate Institute of Child Health, Noida, IND

**Keywords:** hereditary bleeding disorder, hrqol, vwd, hemophilia carrier, hemophilia

## Abstract

Introduction

Women with bleeding disorders continue to be underdiagnosed as well as undertreated. Women with bleeding disorders include those with genotype for hemophilia (traditionally named hemophilia carriers), von Willebrand disease (VWD), platelet function disorders, and rare bleeding disorders. Among these women, the carriers of hemophilia are usually considered asymptomatic. The present study compares the bleeding profile and quality of life (QOL) of women and girls with hemophilia and compares it to those diagnosed with VWD and rare bleeding disorders.

Methods

The present study is part of a prospective observational study (August 2023-July 2028) done on women and girls >12 years in two groups. Group 1 was mothers, sisters, or daughters of patients with hemophilia A who were proven carriers. Group 2 was girls and women registered at our center as following bleeding symptoms and diagnosed as either suffering from VWD or rare bleeding disorders. The bleeding profile was assessed by 1:1 interview using the International Society on Thrombosis and Haemostasis (ISTH) Bleeding Assessment Tool (BAT). Health-related QOL (HRQOL) was assessed using the EuroQOL five dimension *(*EQ5D5L) questionnaire.

Results

The baseline data collected in the first six months of this prospective study is being presented here. At the time of submission, the center caters to 970 patients with hemophilia and inherited bleeding disorders. Eighty girls (post pubertal) and women with either obligate carrier status for hemophilia (n=68) or a previously diagnosed bleeding disorder (10 VWD, one afibrinogenemia, one factor VII deficiency) were enrolled. The median age of Group 1 was 35 years (25-70 years), whereas that of Group 2 was 15.5 years (13-23 years). In Group 1 (women and girls with hemophilia (WGH)), 58 and 10 were carriers of hemophilia A and B, respectively. Additionally, 83% of WGH had more than one family member with bleeding disorder, whereas 75% of Group 2 had a positive family history. The number of family members with the same disorder ranged from 0-4. Among 68 hemophilia carrier women, 20 reported bleeding symptoms (29.4%), of which 18 reported menorrhagia, one antepartum hemorrhage, and one post-partum hemorrhage and two had joint/muscle bleeds and one each had ENT and gastrointestinal bleeding. Three of them were admitted for treatment of excessive bleeding and treated with plasma/red cells. Three women reported gynecological procedures for excessive bleeding, one was on treatment for ovarian cysts, and four received packed red cells after delivery. The BAT score above 6 was reported only in 10% of WGH. Eighteen patients had undergone a surgical procedure in their life, and three of these women required a transfusion during surgery. Only one woman reported similar complaints in her daughter. The comparative analysis of HRQOL showed a significantly worse score for Group 1 in EQ5D5L domains for pain and anxiety/depression compared to that of Group 2.

Conclusions

A significant proportion of previously asymptomatic women with hemophilia carrier status were recognized to have significant bleeding tendencies. The QOL of these carriers is comparable to girls with VWD and rare bleeding disorders and requires special attention.

## Introduction

The narrative that only men bleed in hemophilia is finally changing. Failure to recognize that women can bleed stems from an underestimation of the prevalence of hemophilia in women, a lack of awareness regarding rare bleeding disorders, and difficulties in testing for diseases such as von Willebrand disease (VWD) [1]. Women often trivialize their symptoms, are stigmatized by heavy menstrual bleeding, and often do not vocalize it. There is a growing body of evidence that supports the classification of women with genotype-positive hemophilia as mild, moderate, and severe and as symptomatic and asymptomatic carriers [1]. They are reported to have excessive bleeding and to suffer from poor quality of life (QOL), not just because of their bleeding symptoms. The real-world data on the evaluation of bleeding tendency and quality of life of women with hemophilia is sparse. In this study, we attempt to compare self-reported bleeding tendency and quality of life of previously “asymptomatic” women with hemophilia-genotype with a cohort of girls and women with established bleeding disorders.

This work was presented as a free paper at the World Federation of Hemophilia Congress in 2024 in Madrid.

## Materials and methods

Study site

The present study was conducted in the hemophilia treatment center at the Post Graduate Institute of Child Health, Noida, India. The center at present caters to 970 patients with hemophilia and bleeding disorders from North India, from the states of Uttar Pradesh, Delhi, Bihar, Madhya Pradesh, Uttarakhand, etc. This includes hemophilia A and B, VWD, and rare bleeding disorders, and women with hemophilia genotypes are currently not registered separately.

Study design

The present study is a case-control study conducted in our institute between August 2023 to February 2024. This is the first interim analysis of a 5-year project on the analysis of bleeding phenotype in women with hemophilia.

Study criteria

The inclusion criteria are as follows. The cases (Group 1) are women and girls >12 years (post pubertal) who are obligate carriers or genetically proven carriers of hemophilia. At our center, molecular genetic testing is not done for all patients; hence, only those who were detected to be genotype-positive while being evaluated for carrier status were enrolled. Obligate carriers included daughters of hemophilia patients and women with a brother and one or more sons with hemophilia. The controls (Group 2) are women and girls >12 years who are diagnosed with inherited bleeding disorders such as VWD and rare bleeding disorders, such as factor XIII, X, VII deficiencies, etc. Women who were not hemophilia carriers were excluded from cases.

Ethical consideration

The study received institutional ethics approval prior to initiation (2023-11-IM-62).

Procedure assessment

The enrollment was in order of presentation to the hospital outpatient service. A 1:1 interview lasting 1-1.5 hours each was conducted by our social worker or counsellor. Girls and women in Group 1, being carriers for hemophilia were not proactively evaluated for bleeding before the onset of this study. Those who had divulged bleeding tendencies to us, during routine outpatient visits for their sons, were subsequently evaluated and treated for the same. Group 2 had patients who were >12 years old and already registered with us for established bleeding disorders. They were treated as per standard treatment guidelines such as factor concentrates, tranexamic acid, hormonal therapy for menorrhagia, and oral iron for the prevention and treatment of anemia.

The bleeding profile was assessed using the ISTH Bleeding Assessment Tool [2]. The International Society on Thrombosis and Haemostasis (ISTH) Bleeding Assessment Tool (BAT) score evaluates a history of spontaneous bleeding, such as epistaxis, oral bleeding, bruising, gastrointestinal bleeding, haematuria, menorrhagia, muscle and joint bleeds, intracranial bleeding, bruising, as well as bleeding from minor wounds. It also looks at bleeding after hemostatic challenges such as surgery, post-partum, and tooth extraction. Values less than four in adult males, six in adult females, and three in children were considered normal. Each bleeding symptom was evaluated for the severity and necessity for healthcare facility visits and treatment such as local packing, transfusion, or surgery.

Health-related quality of life (HRQOL) was assessed using the EuroQOL five dimension (EQ5D5L) questionnaire. The Hindi version of EQ5D5L, which was previously validated, was used for the present study [3]. This system analyses QOL under five dimensions: mobility, self-care, usual activities, pain/discomfort, and anxiety/depression. Each of these dimensions is further scored from 1 to 5 as per level of severity. The QOL questionnaire was administered by the counsellor, and the patient’s response was marked as a digit. The final score has five digits for the five dimensions tested. The score also had a visual analogue scale (VAS), which recorded the patient’s self-rated health on a VAS where the endpoints were labelled as “worst health you can imagine” (score 0) to “best health you can imagine” (score 100) on that particular day of the interview. It is used as a quantitative measure of the patient’s self-assessed outcome of her health.

Sample size calculation

As per previous studies reporting 38% positive ISTH BAT score in carriers with hemophilia, a sample size of 363 was calculated based on a confidence interval of 95% and a margin of error of 5% for the entire study spanning five years. The data from the first six months of enrollment is being presented here to highlight the findings that are revelatory.

Statistical analysis

The data were collected and coded into MS Excel (Microsoft® Corp., Redmond, WA) format, and descriptive statistics were described using standard tools. The scores from the BAT score and HRQOL were compared between both cases and controls using the chi-square test and ANOVA as needed.

## Results

The patient enrollment started in December 2023 after ethics approval. Eighty post-pubertal girls and women were enrolled during this initial six-month period of the study. Group 1 constituted 68 women with hemophilia genotype (58 hemophilia A and 10 hemophilia B carriers) who were either proven carriers or obligatory carriers. Group 2 constituted 12 girls/women, with a previously diagnosed bleeding disorder (10 VWD, one afibrinogenemia, one factor VII deficiency). The median age of Group 1 was 35 years (25-70 years), whereas that of Group 2 was 15.5 years (13-23 years).

Eighty-three percent of girls/women in Group 1 had more than one family member with a bleeding disorder, whereas 75% of Group 2 had a positive family history. The number of family members with the same disorder ranged from 0 to 4. Only one woman reported similar complaints of bleeding in her daughter.

Among 68 hemophilia carrier women, 20 reported bleeding symptoms (29.4%), of which 18 reported menorrhagia, one antepartum haemorrhage, one post-abortion bleeding PV, and one post-partum haemorrhage; two had joint/muscle bleeds (one each had ENT and gastrointestinal bleeding); and 3 three of them were admitted for treatment of excessive bleeding and treated with plasma/red cells. No evaluation for bleeding disorders had been done in any of the women diagnosed, despite having a positive family history of hemophilia A. The comparative chart of bleeding profile between the two groups is represented in Table 1 and Table 2.

**Table 1:** Comparison of the bleeding profile of women with hemophilia and other inherited bleeding disorders

Variables	Group A	Group B
Inclusion criteria	Women with hemophilia genotype (HA-58 and HB 10)	Women and girls with vWD and RBD (vWD-10, afibrinogenemia-1, FVII deficiency-1)
Sample size	68	12
Median age (range)	35 years (25-70 years)	15.5 years (13-23 years)
Family history of bleeding disorder	83%	75%
Bleeding symptoms	20/68 (29.4%)	12/12 (100%)
Significant BAT Score >3 adolescents > 6 adult females	7 (10%)	12 (100%)
Hospital admissions for bleeding	3	11
Red cell/ Plasma transfusions	4	10
Gynecological procedures for menorrhagia	3	1
Childbirth	68 underwent childbirth 4 received packed red cells after delivery for anemia	NA

**Table 2:** Sites of bleeding for both groups of patients

Sites of bleeding	Group A	Group B
Menorrhagia	18 (26.4%)	12 (100%)
Antepartum hemorrhage	1 (1.4%)	2 (16.6%)
Postpartum hemorrhage	1 (1.4%)	NA
ENT	1 (1.4%)	4 (33%)
Gastrointestinal hemorrhage	1 (1.4%)	2 (16.6%)
Joint bleeding	0	2 (16.6%)
CNS bleeding	0	2 (16.6%)

Three women reported gynaecological procedures for excessive bleeding, one was on treatment for ovarian cysts, and four received packed red cells after delivery. A BAT score above 6 was reported only in 10% of WGH. Although bleeding tendency was present, since most women did not report it outside their families, they had not received a medical option for the bleeding tendency. Eighteen patients had undergone a surgical procedure in their life and three of these women required a transfusion during the surgery. These surgeries included caesarean section in nine, hysterectomy in one, cervical tear repair in one, tubectomy in two, appendicectomy in one, renal surgery in one, knee replacement in one, cholecystectomy in one, and trauma suturing in one.

The comparative analysis of HRQOL showed comparable QOL for mobility and self-care and significantly worse scores for Group 1 in EQ5D5L domains for usual activities, pain, and anxiety/depression compared to Group 2 (Table 3). The VAS also revealed that 50% of women in Group 1 had a VAS of <80% (Table 4).

**Table 3:** Comparison of quality of life of women with hemophilia and other inherited bleeding disorders

Dimension	Mobility		Self-care		Usual activity		Pain		Anxiety/Depression	
Group	1	2	1	2	1	2	1	2	1	2
Severity										
1	57 (84%)	8 (66.7%)	59 (86%)	9 (75.0%)	46 (67.6%)	5 (41.7%)	36 (53%)	2 (16.7%)	46 (67.6%)	4 (33.3%)
2	4 (5.8%)	3 (25.0%)	5 (7.3%)	2 (16.7%)	14 (20.5%)	3 (25.0%)	15 (22%)	3 (25.0%)	7 (10.2%)	1 (8.3%)
3	1 (1.4%)	0 (0%)	1 (1.4%)	1 (8.3%)	3 (4.4%)	4 (33.3%)	7 (10.2%)	6 (50.0%)	7 (10.2%)	6 (50.0%)
4	3 (4.4%)	1 (8.3%)	2 (3.0%)	0 (0%)	2 (3.0%)	0 (0%)	1 (1.4%)	1 (8.3%)	2 (3.0%)	1 (8.3%)
5	3 (4.4%)	0 (0%)	1 (1.4%)	0 (0%)	1 (1.4%)	0 (0%)	9 (13.2%)	0 (0%)	6 (8.8%)	0 (0%)
P value	0.086		0.123		0.03		0.005		0.014	

**Table 4:** Comparison of quality of life as per visual analogue score of EQ5D5L

Visual analogue score	Group 1	Group 2	Total	P value
<50%	9 (13.2%)	1 (8.3%)	10 (10.7%)	P=0.072
50-80%	26 (38.2%)	9 (75.0%)	35 (45.3%)	
>80%	34 (50%)	2 (16.7%)	33 (44.0%)	
Total	68 (100.0%)	12 (100.0%)	80 (100.0%)	

## Discussion

Hemophilia and bleeding disorders have been long considered to be a condition affecting only boys or men, and women were reported to be “unaffected” or “asymptomatic”. In 2021, as per the ISTH, the new nomenclature recognized that the term hemophilia carrier can hamper clinical recognition and care [4]. The new nomenclature for women categorizes girls and women with factor levels ≤40% as mild moderate and severe and those with >40% level as symptomatic and asymptomatic carriers. The symptoms, in this case, pertain only to bleeding and do not take other issues such as pain or psychosocial difficulties into cognizance. Other than bruising, heavy menstrual bleeding, and bleeding following surgeries, they are known to also have hemorrhagic ovarian cysts, endometriosis, subclinical joint bleeding, and joint damage, which are still missed as they are not linked to the underlying bleeding disorder. The ISTH notes that from a psychological point of view the term “asymptomatic HC” might insufficiently acknowledge the true burden of being a hemophilia carrier; thus, it should be clear that this reflects the bleeding phenotype, not the actual burden for the patient.

In a real-world scenario, there are significant delays in women reporting to a healthcare facility, to be recognized as a potential bleeder and of course in getting adequate treatment. In a survey done in 2016 by our group in which 75 women with bleeding disorders participated globally (Pakistan, USA, Australia, Nigeria, India, Kenya, etc.), 60% reported difficulties in performing household tasks due to their bleeding symptoms [5]. Many reported significant delays in reaching a hospital, as they would often trivialize their symptoms as other women in a family often have it under recognition by physicians and gynecologists. The stigma surrounding the reporting of heavy menstrual bleeding is staggering, and even within families, it has been noted that up to 40% of parents never had a discussion with their own daughters about periods [6]. Women continue to underreport their symptoms and do not present to a hospital facility, thus impeding diagnosis immensely.

It has been estimated that, for every person with hemophilia, there would be around three to five hemophilia carriers in the same family [7]. About 30% of these women will have low factor levels, and they should thus constitute around 30% of the hemophilia men diagnosed at any center [7].

In our cohort, out of 912 hemophilia patients, around 600 women are anticipated to be carriers (genotype-positive hemophilia); out of them, 182 should be symptomatic since the factor level is expected to be less than 40%. However, none of these women (sisters, mothers, or daughters of patients with hemophilia) have reported to us bleeding symptoms despite them accompanying the index patient to the hospital on multiple visits. With this hypothesis in mind, this study was planned to proactively look for bleeding symptoms and QOL in this cohort of proven/obligate carriers of hemophilia and compare them against an established cohort of girls/women with severe bleeding disorders such as VWD or rare bleeding disorders. 

Almost one-third of our cohort of women with hemophilia genotype had a bleeding phenotype that was previously unrecognized. Although treated for bleeding with transfusions and even surgical procedures such as hysterectomy, none of these women had been tested for a bleeding disorder, despite having borne sons with hemophilia. This highlights the gross under-recognition of their bleeding phenotype and the need to institute emergent action pathways to facilitate their diagnosis. Although factor assay is not available for most at present, this is in line with the expectation of a bleeding phenotype in one-third of women with hemophilia. In addition, we wish to highlight the missed opportunities in the form of 18 major surgeries and 68 childbirth, which could have been evaluated for haemostatic adequacy.

In a previous study, evaluating 168 hemophilia carriers, 65 (38%) had a positive or abnormal bleeding score, with a mean score of 5.7 in carriers against 1.43 in controls [8]. In our cohort, although bleeding symptoms were noted in around 30% of the proven/obligate carriers, this was not reflected in the ISTH BAT score. A positive BAT score of >6 was reported only by 10% of these women; this could be because of the fact that, even with bleeding, many would not report to a hospital or seek any form of medical treatment. Often these women feel their symptoms are trivial compared to their sons or brothers and often they do not want to pressurize the family further by demanding medical help for themselves. Many would also not want to divulge their carrier state to the medical facility at the time of childbirth or other surgical interventions due to the stigma associated with the carrier state and its psychosocial challenges.

The other aspect of the study is to assess the quality of this cohort of women. The dimensions of QOL were comparable in three domains (mobility and self-care) and poorer in three domains (usual activities, pain, and anxiety/depression) in women with hemophilia genotype, compared to their bleeding counterparts. This also highlights that, in addition to bleeding, there are other factors that drive the impact on HRQOL. This could be anemia, pain in the lower back and joints, or subclinical joint pain due to possible soft tissue changes, which have been noted even in women who have factor levels >40% [9].

We propose to develop an adaptive care pathway for girls and women with bleeding, considering the different challenges encountered in different periods of life. In hemophilia care, the concept of anticipatory guidance has proved to be very effective [10]. In women with bleeding, the path to a correct and timely diagnosis is riddled with issues [11]. Once diagnosed, the tribulations faced at different ages while raising a child with a bleeding disorder are similar; thus, timely counseling can help families "anticipate" difficulties and take preventive and remedial steps. Similarly, in women, understanding the lifecycle of a woman with bleeding can help us develop guidance for what to expect during childhood, the timing of carrier detection, adolescence with the onset of menarche, management options for heavy menstrual bleeding, supplementation of iron for prevention of anemia, adulthood with challenges of sexuality, fertility, pregnancy and childbirth, adoption of protocol-based management for deliveries for both mother and child, prevention and treatment of post-partum hemorrhage, detection of joint status and osteoporosis, options for contraception, and preventive healthcare for elderly and management of menopause [1,12]. This will also help us standardize care with the advent of innovative treatment options in the care of bleeding disorders. Additionally, it is crucial to “destigmatize” carrier detection so that families come forward for it not only from a prenatal testing perspective but also for the health of the hemophilia carrier. We also encourage patient organizations catering to bleeding disorders to openly discuss heavy menstrual bleeding and other bleeding-related issues in women, to reduce the shame and stigma associated with it. This will encourage more women to report their symptoms and seek timely medical help.

The limitation of our study is that it has not tested the factor level of these women and thus a definite classification will not be possible at this stage of the study. Similarly, a detailed clinical and laboratory evaluation of bleeding such as degree of anemia, level of ferritin, and detailed musculoskeletal assessments is beyond the scope of this current work. However, they are being planned as part of a prospective study with funding support. The recognition of this bleeding phenotype is important and will pave the path for better follow-up of this cohort.

## Conclusions

A significant proportion of previously asymptomatic women with hemophilia carrier status were recognized to have significant bleeding tendencies. The QOL of these carriers is comparable to girls with VWD and rare bleeding disorders and requires special attention. The study clearly demonstrates the need for actively searching for bleeding, as well as other medical issues in women who are hemophilia carriers. These silent invisible mothers and sisters of hemophilic boys/men need to be recognized, and the focus has to be diverted to improving the quality of life to be integrated into their medical services.
